# Supplementary figures and images for: Changes in social contacts in England during the COVID-19 pandemic between March 2020 and March 2021 as measured by the CoMix survey: A repeated cross-sectional study
Source: PLoS Med. 2022 Mar 1;19(3):e1003907. doi: 10.1371/journal.pmed.1003907 (PMC8887739; doi:10.1371/journal.pmed.1003907)

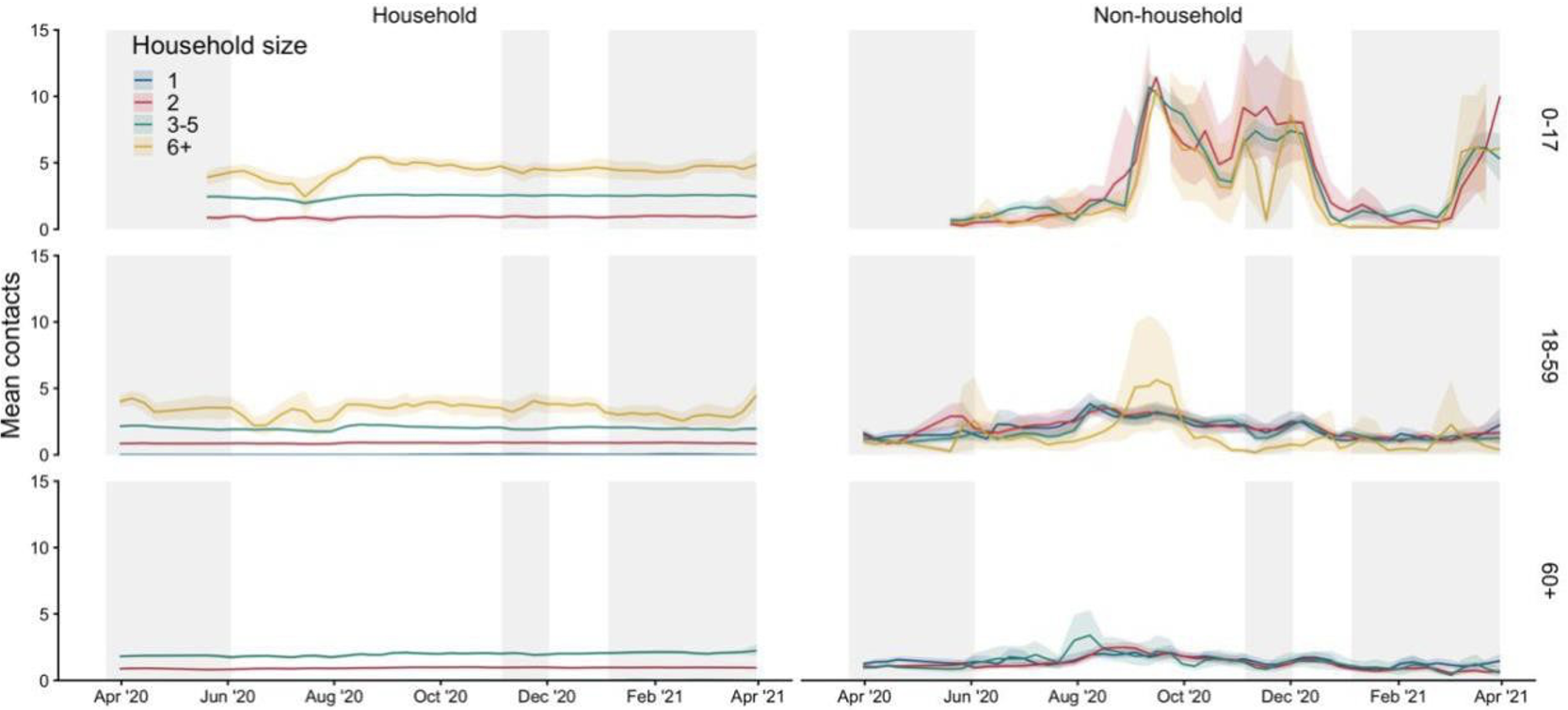

Supplement: S1 Fig — Bootstrapped means weighted by age, gender, and weekday. Households of 6 or more for the 60 and older age group was omitted due to a low number of participants in the category. (TIF) [file pmed.1003907.s001.tif]

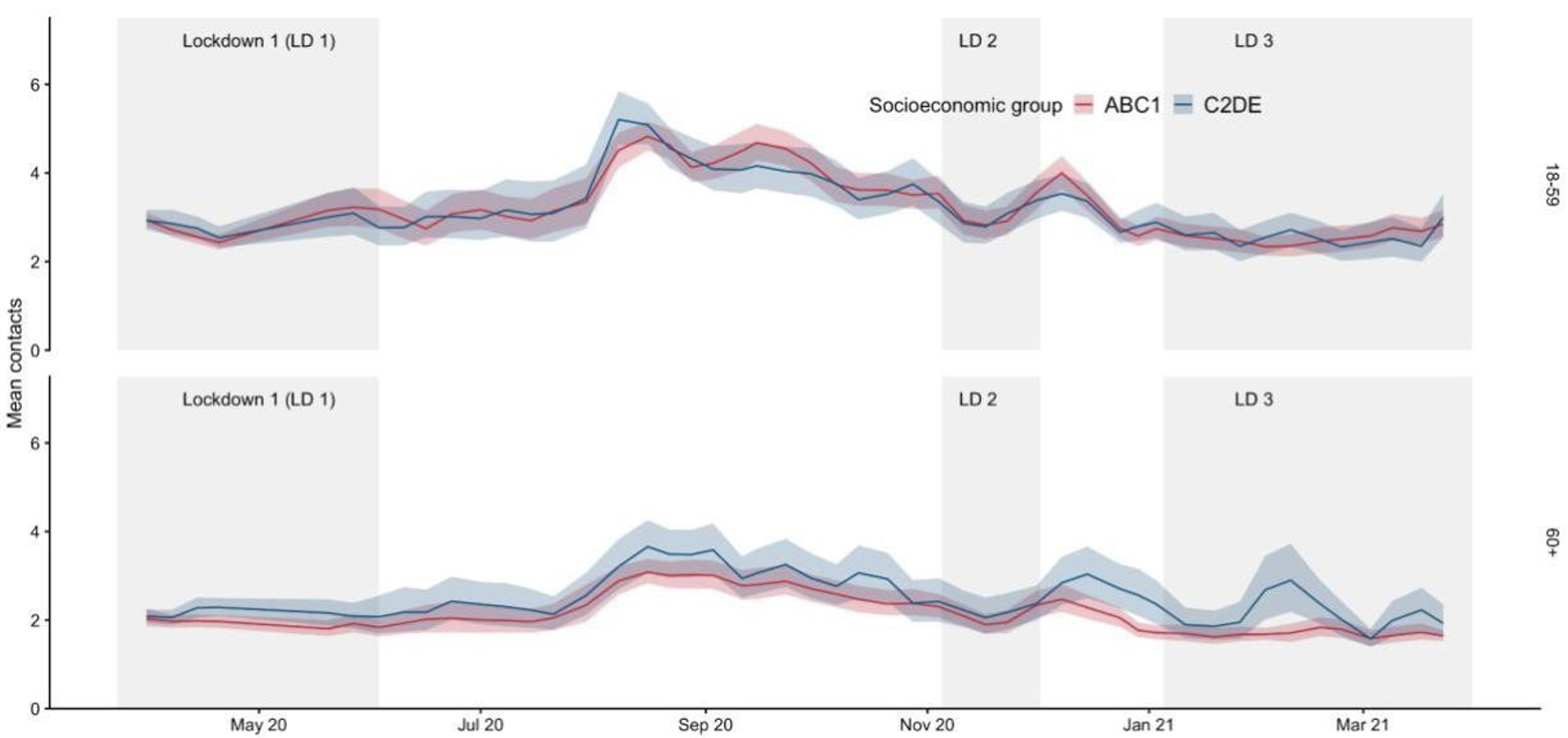

Supplement: S2 Fig — Bootstrapped mean contacts of participants weighted by age, gender, and weekday. Social groups ABC1 include the socioeconomic categories A, B, and C1 and the social groups C2DE include the socioeconomic categories C2, D, and E as shown by occupation (see S3 Text). CI, confidence interval. (TIF) [file pmed.1003907.s002.tif]

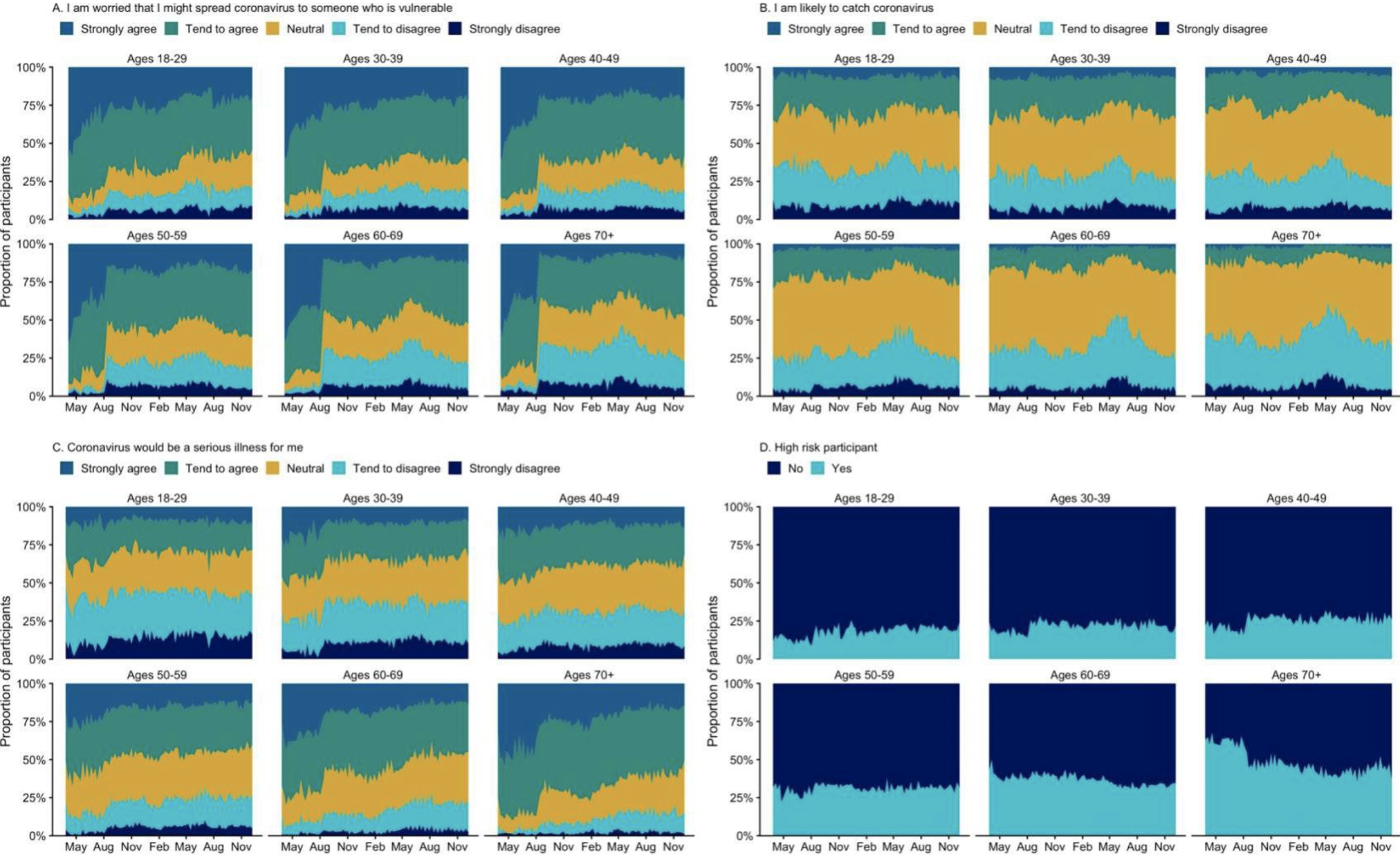

Supplement: S3 Fig — The raw proportion of Likert scale responses and self-reported risk status among adult participants. (TIF) [file pmed.1003907.s003.tif]

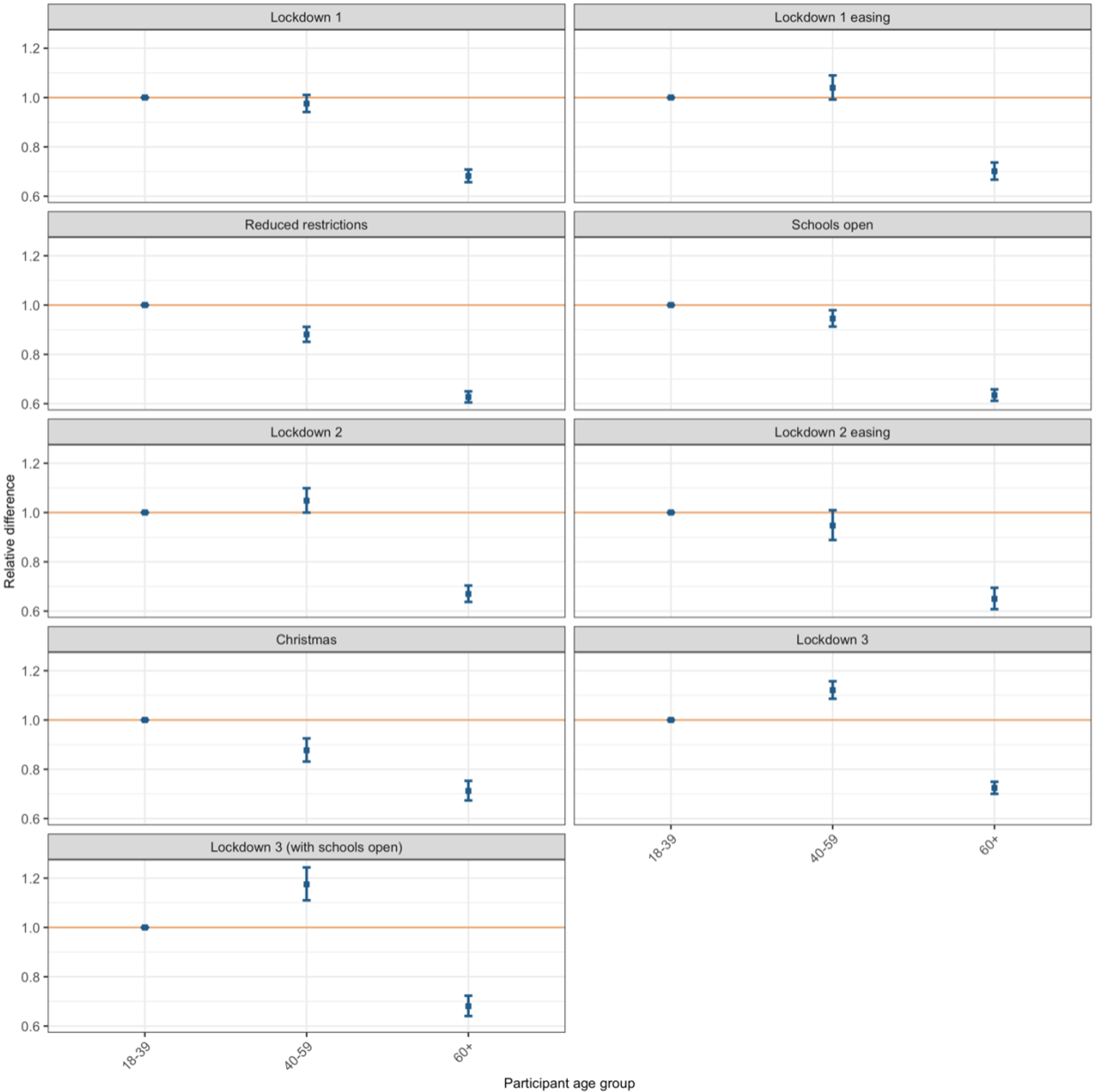

Supplement: S4 Fig — Relative differences calculated using a GAM with participants aged 18 to 39 as the reference period for each age group adjusted to the UK population by age and gender (when available) for the age groups 40 to 59 and 60+ years old. CI, confidence interval; GAM, generalised additive model. (TIF) [file pmed.1003907.s004.tif]

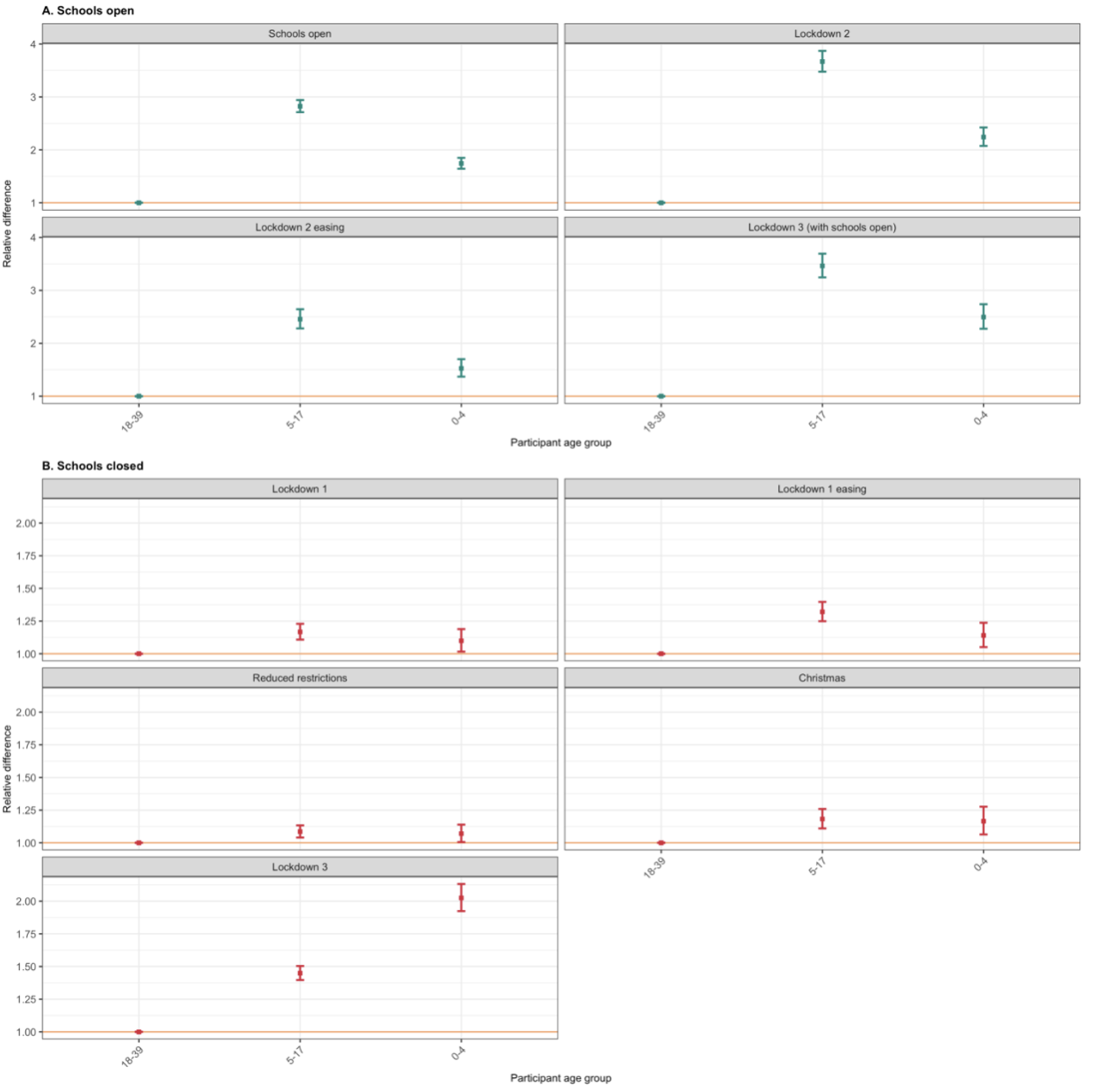

Supplement: S5 Fig — Relative differences calculated using a GAM with participants aged 18 to 39 as the reference period for each age group adjusted to the UK population by age and gender (when available) for the age groups 0 to 4 and 5 to 17 years old. (A) Study periods in which schools were open. (B) Study periods in which schools were closed. CI, confidence interval; GAM, generalised additive model. (TIF) [file pmed.1003907.s005.tif]

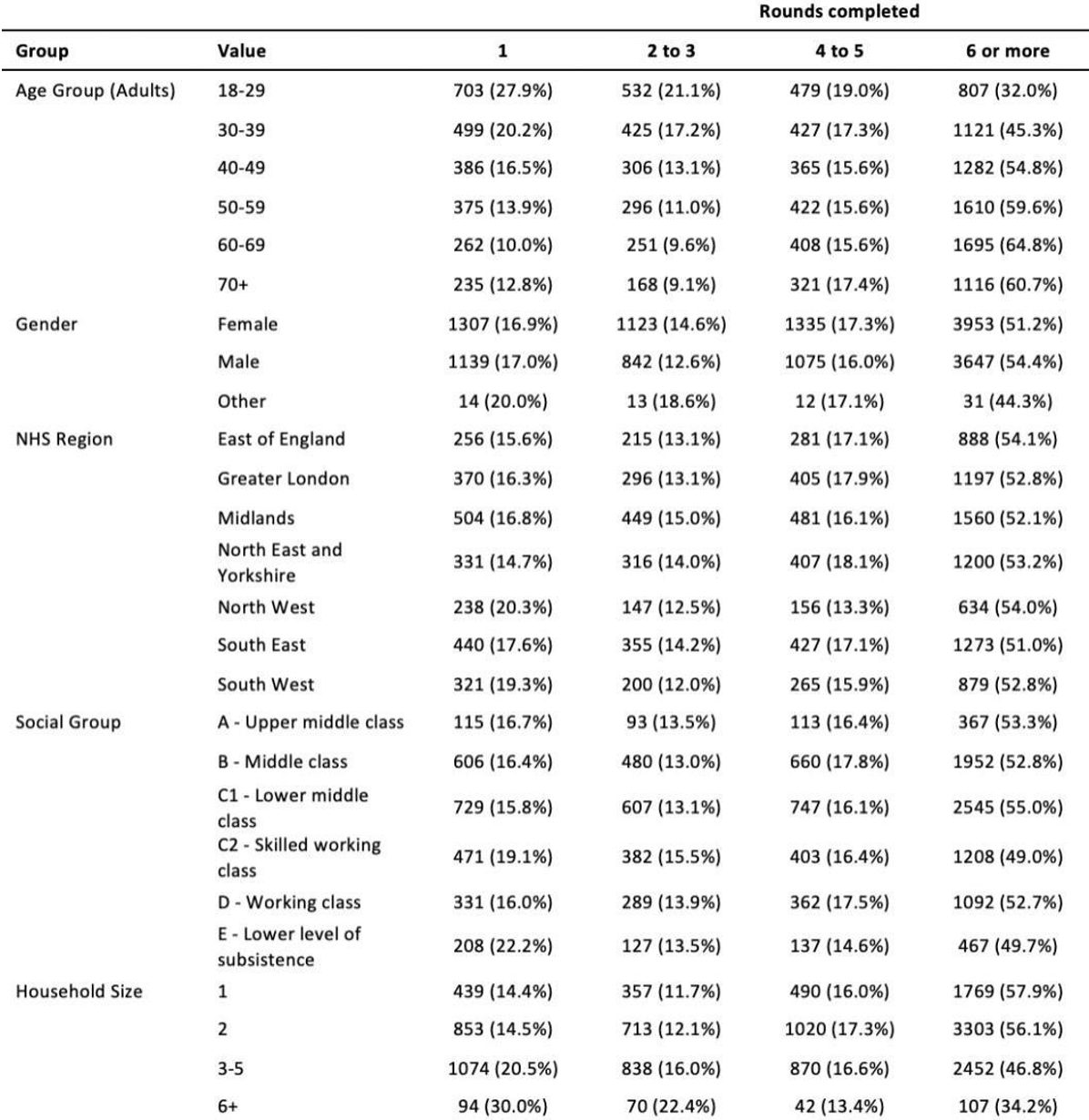

Supplement: S1 Table — NHS, National Health Service. (TIF) [file pmed.1003907.s007.tif]

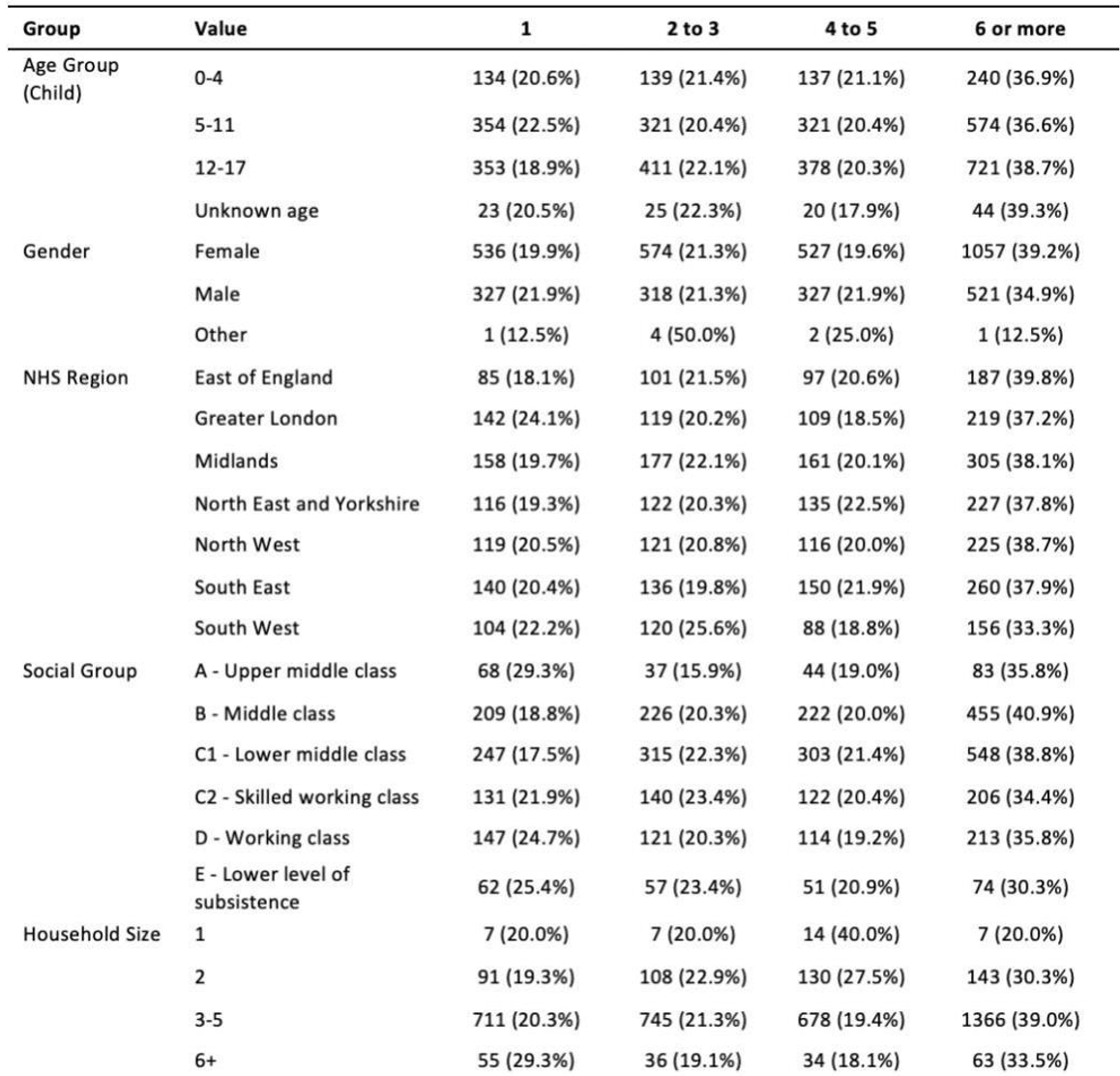

Supplement: S2 Table — Parents of children report gender by answering the question “As far as you know, which of the following describes how [NAME OF CHILD] thinks of themselves?,” with the options “Male,” “Female,” “In another way,” “Do not know,” and “Prefer not to answer.” NHS, National Health Service. (TIF) [file pmed.1003907.s008.tif]

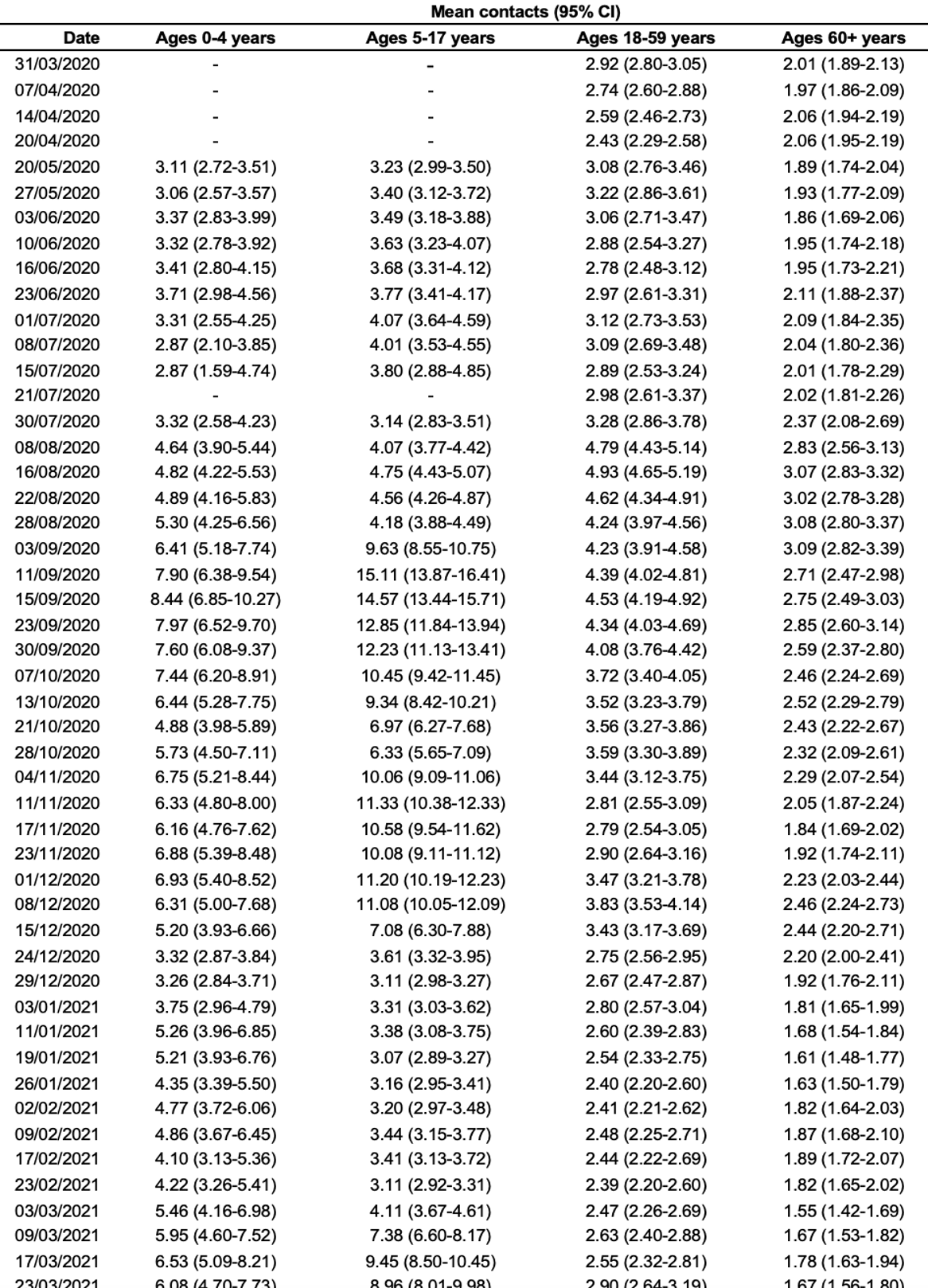

Supplement: S3 Table — Mean reported contacts of participants weighted by age, gender, and weekday. CI, confidence interval. (TIF) [file pmed.1003907.s009.tif]

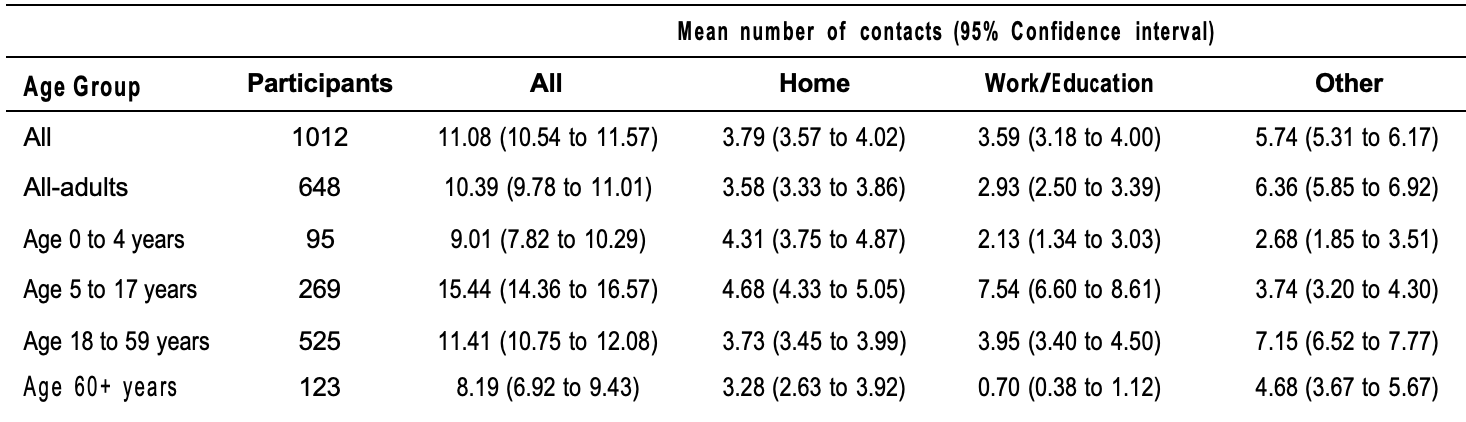

Supplement: S4 Table — Reported by age groups of 0 to 4, 5 to 17, 18 to 59, and 60 or more year, weighted by day of week. Participants were able to report the same contact in multiple settings. The number of participants in each age group is included. CI, confidence interval. (TIF) [file pmed.1003907.s010.tif]
