# Supplementary material for: Changes in social contacts in England during the COVID-19 pandemic between March 2020 and March 2021 as measured by the CoMix survey: A repeated cross-sectional study
Source: PLoS Med. 2022 Mar 1;19(3):e1003907. doi: 10.1371/journal.pmed.1003907 (PMC8887739; doi:10.1371/journal.pmed.1003907)
Supplement: S6 Fig — (A) Contact matrices for all contacts in England for Lockdown 1, Lockdown 1 easing, and Relaxed restrictions (Diagonal) and the element-wise absolute difference between the matrices (off diagonal). Contacts censored to 50 contacts per participant. Lockdown 1 data from March 23 to June 3, 2020 and Lockdown 3 data from January 5 to 18, 2021. (B) Contact matrices for all contacts in England for Schools reopening, Lockdown 2 and Lockdown 2 easing (Diagonal), and the element-wise absolute difference between the matrices (off diagonal). Contacts censored to 50 contacts per participant. Lockdown 1 data from March 23 to June 3, 2020 and Lockdown 3 data from January 5 to 18, 2021. (C) Contact matrices for all contacts in England for Christmas, Lockdown 3 and Lockdown 3 easing (Diagonal), and the element-wise absolute difference between the matrices (off diagonal). Contacts censored to 50 contacts per participant. Lockdown 1 data from March 23 to June 3, 2020 and Lockdown 3 data from January 5 to 18, 2021. (PDF) [file pmed.1003907.s006.pdf]

S6 Figure: Contact matrices with absolute differences by time period. A) Contact matrices for all contacts in England for Lockdown 1, Lockdown 1 easing and Relaxedrestrictions (Diagonal) and the element-wise absolute difference between the matrices (off diagonal). Contacts censored to 50 contacts per participant. Lockdown 1 data from 23rd of March to 3rd of June2020Lockdown 3 data from 5th to 18th of January 2021.; **B)** Contact matrices for all contacts in England for Schools reopening, Lockdown 2 and Lockdown 2 easing (Diagonal) and the element-wise absolute difference between the matrices (off diagonal). Contacts censored to 50 contacts per participant. Lockdown 1 data from 23rd of March to 3rd of June 2020 Lockdown 3 data from 5th to 18th of January 2021; **C)** Contact matrices for all contacts in England for Christmas, Lockdown 3 and Lockdown 3 easing (Diagonal) and the element-wise absolute difference between the matrices (off diagonal). Contacts censored to 50 contacts per participant. Lockdown 1 data from 23rd of March to 3rd of June 2020 Lockdown 3 data from 5th to 18th of January 2021.

Contact matrices

A)

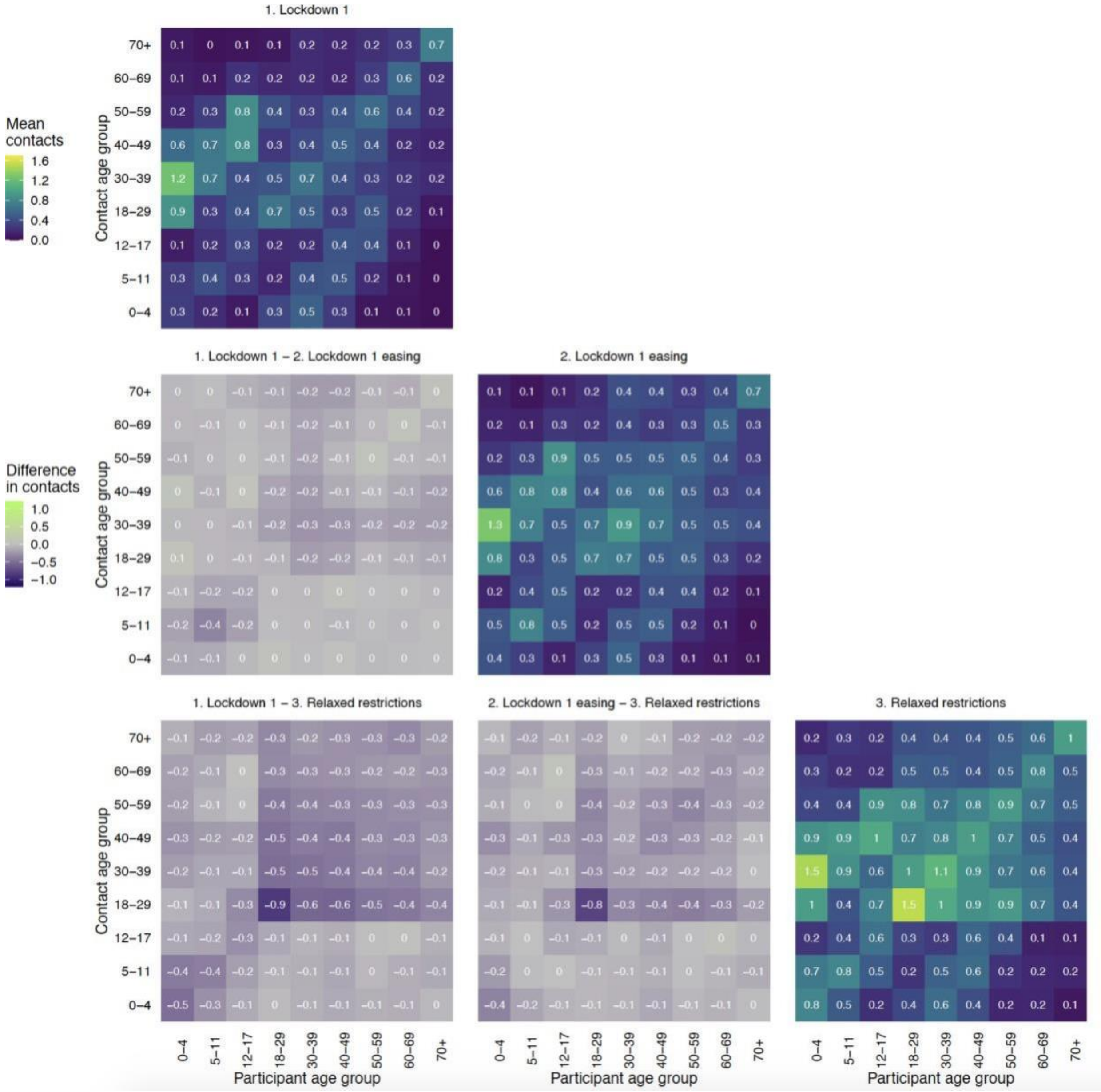

B)

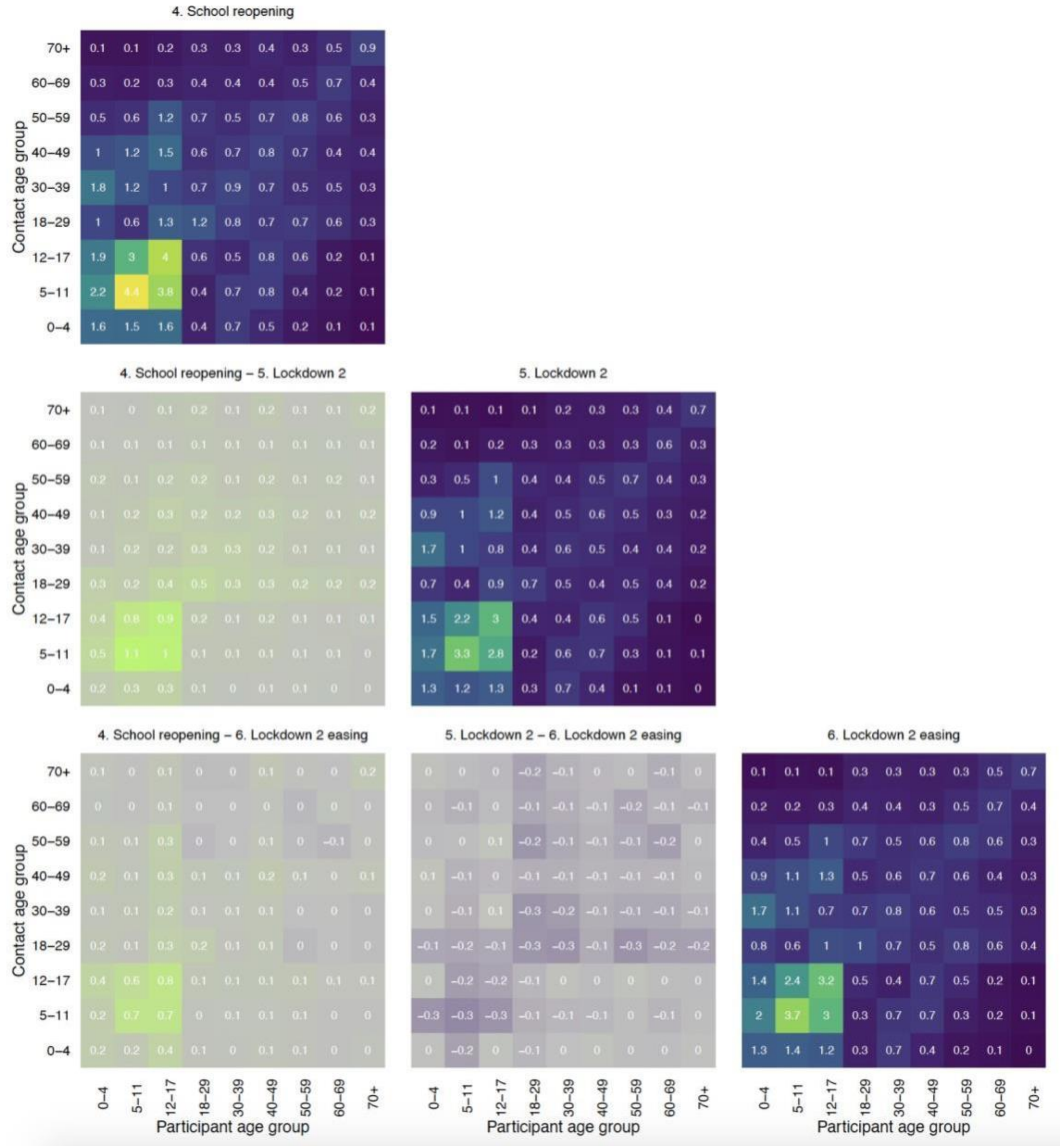

4. School reopening
Mean contacts
4. School reopening – 5. Lockdown 2
5. Lockdown 2
Difference in contacts
4. School reopening – 6. Lockdown 2 easing
5. Lockdown 2 – 6. Lockdown 2 easing
6. Lockdown 2 easing
Contact age group
Participant age group

C)

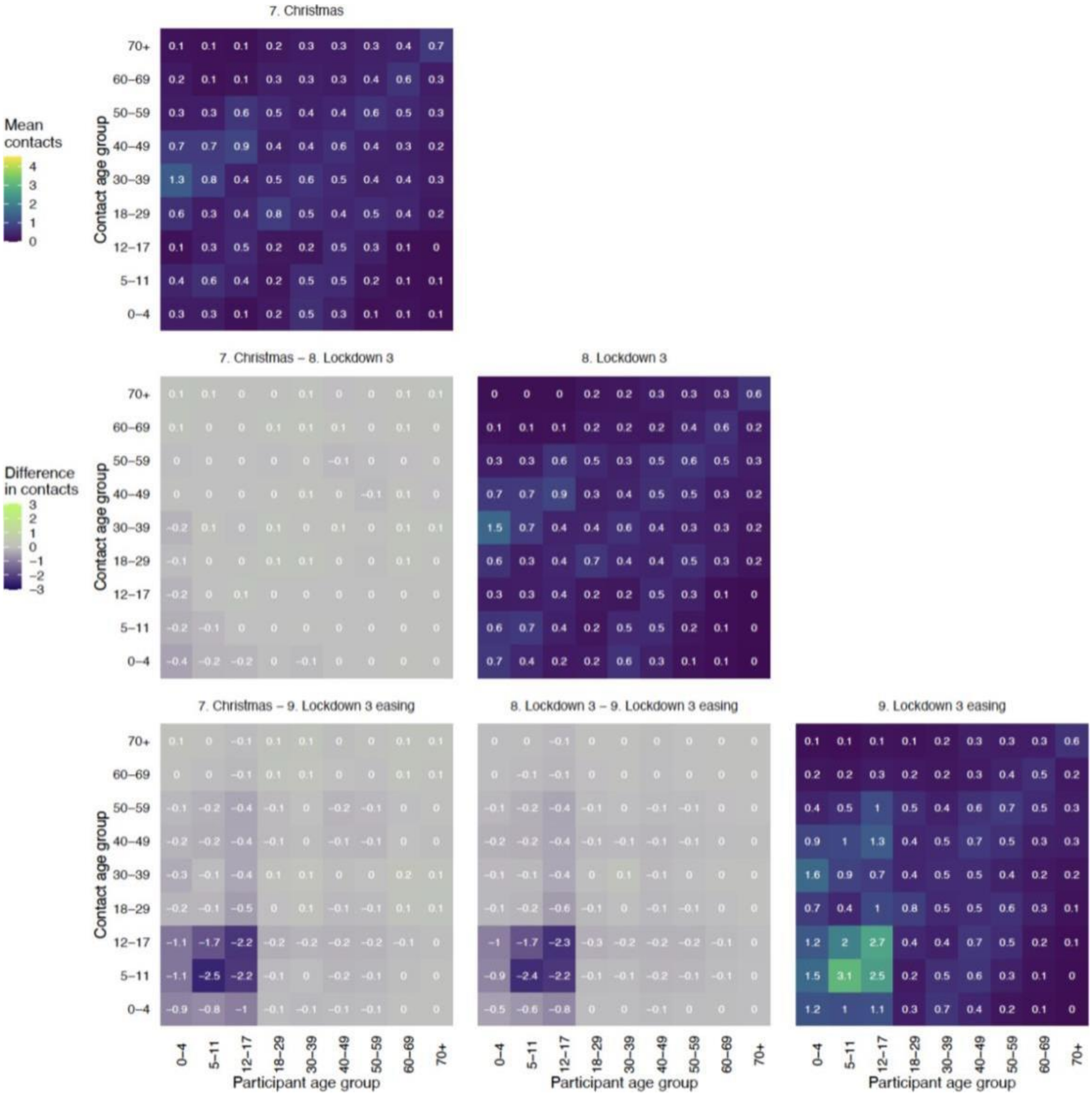

7. Christmas
Mean contacts
Contact age group
7. Christmas – 8. Lockdown 3
8. Lockdown 3
Difference in contacts
7. Christmas – 9. Lockdown 3 easing
8. Lockdown 3 – 9. Lockdown 3 easing
9. Lockdown 3 easing
Participant age group
0–4
5–11
12–17
18–29
30–39
40–49
50–59
60–69
70+
