# Supplementary material for: Changes in social contacts in England during the COVID-19 pandemic between March 2020 and March 2021 as measured by the CoMix survey: A repeated cross-sectional study
Source: PLoS Med. 2022 Mar 1;19(3):e1003907. doi: 10.1371/journal.pmed.1003907 (PMC8887739; doi:10.1371/journal.pmed.1003907)
Supplement: S3 Text — Recruitment methods and social category mapped to reported occupations. (PDF) [file pmed.1003907.s013.pdf]

Survey Recruitment

Data was collected on behalf of our study team by Ipsos, an international market research company. Participants are recruited by Ipsos from a variety of sources to create panels that are representative of the population in which they are recruited. Ipsos primarily recruits through social networks, allowing them to target hard to recruit populations, and includes providing participant-relevant incentives for completing surveys. Other methods for recruitment include email lists, banners, website and text ads, co-registration, and search engine marketing. When necessary, they also partner with thoroughly vetted third party recruiters. Ipsos limits the number of surveys each participant is able to complete in a given time period and uses algorithms to detect fraud and remove users from the survey in real-time. For this survey, Ipsos recruited adults (ages 18 years and older) in census representative age bands. We compare our participants to census figures by age, gender, and household size

Ipsos-MORI occupation to social grade map

Main Earner Occupation	SOCIAL GRADE
1100. Legislators and senior officials	1. A - Upper middle class
1210. Directors and chief executives	1. A - Upper middle class
1221. Production and operations department managers in agriculture, hunting, forestry and fishing	3. C1 - Lower middle class
1222. Production and operations department managers in manufacturing	3. C1 - Lower middle class
1223. Production and operations department managers in construction	3. C1 - Lower middle class
1224. Production and operations department managers in wholesale and retail trade	3. C1 - Lower middle class
1225. Production and operations department managers in restaurants and hotels	3. C1 - Lower middle class
1226. Production and operations department managers in transport, storage and communications	3. C1 - Lower middle class
1227. Production and operations department managers in business services	3. C1 - Lower middle class
1228. Production and operations department managers in personal care, cleaning and related services	3. C1 - Lower middle class
1229. Production and operations department managers not elsewhere classified	3. C1 - Lower middle class
1231. Finance and administration department managers	2. B - Middle class
1232. Personnel and industrial relations department managers	2. B - Middle class
1233. Sales and marketing department managers	2. B - Middle class
1234. Advertising and public relations department managers	2. B - Middle class
1235. Supply and distribution department managers	2. B - Middle class
1236. Computing services department managers	2. B - Middle class
1237. Research and development department managers	2. B - Middle class
1239. Other department managers not elsewhere classified	2. B - Middle class
1311. General managers in agriculture, hunting, forestry/ and fishing	2. B - Middle class
1312. General managers in manufacturing	2. B - Middle class
1313. General managers in construction	2. B - Middle class
1314. General managers in wholesale and retail trade	2. B - Middle class
1315. General managers of restaurants and hotels	2. B - Middle class
1316. General managers in transport, storage and communications	2. B - Middle class
1317. General managers of business services	2. B - Middle class
1318. General managers in personal care, cleaning and related services	2. B - Middle class
1319. General managers not elsewhere classified	2. B - Middle class
2110. Physicists, chemists and related professionals	2. B - Middle class
2120. Mathematicians, statisticians and related professionals	2. B - Middle class
2130. Computing professionals	2. B - Middle class
2141. Architects, town and traffic planners	2. B - Middle class
2142. Civil engineers	2. B - Middle class

2143. Electrical engineers	2. B - Middle class
2144. Electronics and telecommunications engineers	2. B - Middle class
2145. Mechanical engineers	2. B - Middle class
2146. Chemical engineers	2. B - Middle class
2147. Mining engineers, metallurgists and related professionals	2. B - Middle class
2148. Cartographers and surveyors	2. B - Middle class
2149. Architects, engineers and related professionals not elsewhere classified	2. B - Middle class
2210. Life science professionals	2. B - Middle class
2220. Health professionals (except nursing)	2. B - Middle class
2230. Nursing and midwifery professionals	3. C1 - Lower middle class
2300. Teaching professionals	3. C1 - Lower middle class
2410. Business professionals	2. B - Middle class
2420. Legal professionals	2. B - Middle class
2430. Archivists, librarians and related information professionals	3. C1 - Lower middle class
2440. Social science and related professionals	2. B - Middle class
2450. Writers and creative or performing artists	2. B - Middle class
2460. Religious professionals	2. B - Middle class
3110. Physical and engineering science technicians	3. C1 - Lower middle class
3120. Computer associate professionals	3. C1 - Lower middle class
3130. Optical and electronic equipment operators	3. C1 - Lower middle class
3140. Ship and aircraft controllers and technicians	3. C1 - Lower middle class
3150. Safety and quality inspectors	4. C2 - Skilled working class
3200. Life science and health associate professionals	3. C1 - Lower middle class
3300. Teaching associate professionals	3. C1 - Lower middle class
3410. Finance and sales associate professionals	3. C1 - Lower middle class
3420. Business services agents and trade brokers	3. C1 - Lower middle class
3430. Administrative associate professionals	4. C2 - Skilled working class
3440. Customs, tax and related government associate professionals	3. C1 - Lower middle class
3450. Police inspectors and detectives	2. B - Middle class
3460. Social work associate professionals	4. C2 - Skilled working class
3470. Artistic, entertainment and sports associate professionals	3. C1 - Lower middle class
3480. Religious associate professionals	3. C1 - Lower middle class
4100. Office clerks	3. C1 - Lower middle class
4200. Customer services clerks	3. C1 - Lower middle class
5110. Travel attendants and related workers	3. C1 - Lower middle class
5120. Housekeeping and restaurant services workers	5. D - Working class
5130. Personal care and related workers	5. D - Working class
5140. Other personal services workers	5. D - Working class
5160. Protective services workers	3. C1 - Lower middle class
5200. Models, salespersons and demonstrators	4. C2 - Skilled working class
6000. Skilled agricultural and fishery workers	5. D - Working class
7100. Extraction and building trades workers	4. C2 - Skilled working class
7210. Metal moulders, welders, sheet-metal workers, structural - metal preparers, and related trades workers	4. C2 - Skilled working class

7220. Blacksmiths, tool-makers and related trades workers	4. C2 - Skilled working class
7230. Machinery mechanics and fitters	4. C2 - Skilled working class
7240. Electrical and electronic equipment mechanics and fitters	4. C2 - Skilled working class
7310. Precision workers in metal and related materials	4. C2 - Skilled working class
7320. Potters, glass-makers and related trades workers	4. C2 - Skilled working class
7330. Handicraft workers in wood, textile, leather and related materials	4. C2 - Skilled working class
7340. Printing and related trades workers	4. C2 - Skilled working class
7410. Food processing and related trades workers	4. C2 - Skilled working class
7420. Wood treaters, cabinet-makers and related trades workers	4. C2 - Skilled working class
7430. Textile, garment and related trades workers	4. C2 - Skilled working class
7440. Pelt, leather and shoemaking trades workers	4. C2 - Skilled working class
8000. Plant and machine operators and assemblers	5. D - Working class
9100. Sales and services elementary occupations	5. D - Working class
9200. Agricultural, fishery and related labourers	5. D - Working class
9300. Labourers in mining, construction, manufacturing and transport	5. D - Working class
9888. Armed forces	3. C1 - Lower middle class
9991. Unemployed and not looking for a job / Long-term sick or disabled	6. E - Lower level of subsistence
9992. Pupil /Student/ in full time education	6. E - Lower level of subsistence
9993. Housewife	6. E - Lower level of subsistence
