# Supplementary material for: Changes in social contacts in England during the COVID-19 pandemic between March 2020 and March 2021 as measured by the CoMix survey: A repeated cross-sectional study
Source: PLoS Med. 2022 Mar 1;19(3):e1003907. doi: 10.1371/journal.pmed.1003907 (PMC8887739; doi:10.1371/journal.pmed.1003907)
Supplement: S4 Text — Checklist of items that should be included in reports of cross-sectional studies. STROBE, STrengthening the Reporting of OBservational studies in Epidemiology. (PDF) [file pmed.1003907.s014.pdf]

STROBE Statement—Checklist of items that should be included in reports of ***cross-sectional studies***

	Item No	Recommendation	Line No
**Title and abstract**	1	(*a*) Indicate the study's design with a commonly used term in the title or the abstract	Title/Abstract Introduction P1
		(*b*) Provide in the abstract an informative and balanced summary of what was done and what was found	Abstract Methods and findings P1
**Introduction**			
Background/rationale	2	Explain the scientific background and rationale for the investigation being reported	Introduction P1/P2
Objectives	3	State specific objectives, including any prespecified hypotheses	Introduction P4
Methods			
Study design	4	Present key elements of study design early in the paper	Methods – *Study design* P1-P5
Setting	5	Describe the setting, locations, and relevant dates, including periods of recruitment, exposure, follow-up, and data collection	Methods – *Study design* P1-P3
Participants	6	(*a*) Give the eligibility criteria, and the sources and methods of selection of participants	Methods – *Study design* P1-P3
Variables	7	Clearly define all outcomes, exposures, predictors, potential confounders, and effect modifiers. Give diagnostic criteria, if applicable	Methods – *Survey design* P1-P3
Data sources/ measurement	8*	For each variable of interest, give sources of data and details of methods of assessment (measurement). Describe comparability of assessment methods if there is more than one group	Methods – *Survey design* P1-P3
Bias	9	Describe any efforts to address potential sources of bias	Methods – *Statistical Analysis Plan* – P2-P5
Study size	10	Explain how the study size was arrived at	Methods – *Study design* P4
Quantitative variables	11	Explain how quantitative variables were handled in the analyses. If applicable, describe which groupings were chosen and why	Methods – *Statistical Analysis Plan* – P3-P6, P8-12
Statistical methods	12	(*a*) Describe all statistical methods, including those used to control for confounding	Methods – *Statistical Analysis Plan* – P1-P12
		(*b*) Describe any methods used to examine subgroups and interactions	Methods – *Statistical Analysis Plan* – P4-P12
		(*c*) Explain how missing data were addressed	Methods – *Statistical Analysis Plan* – P4

		(*d*) If applicable, describe analytical methods taking account of sampling strategy	Methods – *Statistical Analysis Plan* – P3-P6, P8-P9
		(*e*) Describe any sensitivity analyses	Methods – *Statistical Analysis Plan* – P5
**Results**			
Participants	13*	(a) Report numbers of individuals at each stage of study—eg numbers potentially eligible, examined for eligibility, confirmed eligible, included in the study, completing follow-up, and analysed	Results – *Participant characteristics* - P1-P3
		(b) Give reasons for non-participation at each stage	Results – *Participant characteristics* - P1
		(c) Consider use of a flow diagram	No diagram - Tables S1, S2
Descriptive data	14*	(a) Give characteristics of study participants (eg demographic, clinical, social) and information on exposures and potential confounders	Results – *Participant characteristics* - P1-P3
		(b) Indicate number of participants with missing data for each variable of interest	NA
Outcome data	15*	Report numbers of outcome events or summary measures	Results – *Mean contacts…* P1-P2
Main results	16	(*a*) Give unadjusted estimates and, if applicable, confounder-adjusted estimates and their precision (eg, 95% confidence interval). Make clear which confounders were adjusted for and why they were included	Results – *Mean contacts…* P1-P2
		(*b*) Report category boundaries when continuous variables were categorized	Methods – *Statistical Analysis Plan* – P11<br>Results - P2
		(*c*) If relevant, consider translating estimates of relative risk into absolute risk for a meaningful time period	NA
Other analyses	17	Report other analyses done—eg analyses of subgroups and interactions, and sensitivity analyses	Results – *Mean contacts…* P3-P14
**Discussion**			
Key results	18	Summarise key results with reference to study objectives	Discussion P1
Limitations	19	Discuss limitations of the study, taking into account sources of potential bias or imprecision. Discuss both direction and magnitude of any potential bias	Discussion – *Limitations* – P1
Interpretation	20	Give a cautious overall interpretation of results considering objectives, limitations, multiplicity of analyses, results from similar studies, and other relevant evidence	Discussion – *Comix in Context* – P1-P2<br>Discussion – *Conclusions* – P1

Generalisability	21	Discuss the generalisability (external validity) of the study results	Discussion – *Limitations* – P1
**Other information**			
Funding	22	Give the source of funding and the role of the funders for the present study and, if applicable, for the original study on which the present article is based	Funding statement

*Give information separately for exposed and unexposed groups.

Note: An Explanation and Elaboration article discusses each checklist item and gives methodological background and published examples of transparent reporting. The STROBE checklist is best used in conjunction with this article (freely available on the Web sites of PLoS Medicine at http://www.plosmedicine.org/, Annals of Internal Medicine at http://www.annals.org/, and Epidemiology at http://www.epidem.com/). Information on the STROBE Initiative is available at www.strobe-statement.org.
