# Supplementary material for: Changes in social contacts in England during the COVID-19 pandemic between March 2020 and March 2021 as measured by the CoMix survey: A repeated cross-sectional study
Source: PLoS Med. 2022 Mar 1;19(3):e1003907. doi: 10.1371/journal.pmed.1003907 (PMC8887739; doi:10.1371/journal.pmed.1003907)
Supplement: Attachment — Submitted filename: Reviewer_editor_feedback_and_response_submission3.docx [file pmed.1003907.s016.docx]

Dear Editors,

I would like to thank the editors and all three reviewers for their constructive feedback on the original manuscript. I have added comments below to address most of the comments, which indicate all updates to the manuscript.

I have been able to address most of the editor and reviewer feedback with some minor exceptions due to technical limitations, noted below.

If there are any questions, please do not hesitate to contact me as needed.

Best wishes,

Amy

**Feedback with response:**

Requests from the editors:

From the Academic Editor:

1. Abstract: Abstract: Methods and Findings: The sentence “The mean of reported contacts among adults have reduced compared to previous surveys with adults aged 18 to 59 reporting a mean of 2.39 (95% CI 2.20 - 2.60) contacts to 4.93 (95% CI 4.65 - 5.19) contacts, and the mean contacts for school-age children was 3.07 (95% CI 2.89 - 3.27) to 15.11 (95% CI 13.87 - 16.41).” is confusing. Since previous surveys are mentioned, please provide some quantitative information on how they compare to these results.

**Updated to :**
The mean reported contacts for adults 18 to 59 years old ranged between 2.39 (95% CI 2.20 - 2.60) contacts and 4.93 (95% CI 4.65 - 5.19) contacts during the study period. The mean contacts for school-age children (5-17 years old) ranged from 3.07 (95% CI 2.89 - 3.27) to 15.11 (95% CI 13.87 - 16.41). This demonstrates a sustained decrease in social contacts compared to a mean of 11.7 contacts per participant measured by the POLYMOD social contact study in 2005/06.

2. Abstract: Conclusions: Line 37-38: “...” There is no data provided in the abstract on pre-pandemic levels and one must assume that the authors mean in reference to the Polymod study although this is not stated until much later in the paper.

**I have added more references to POLYMOD earlier in the text**

3. Results: Contacts by Setting: Page 11: One would not expect household size to change due to Covid so it would be useful to compare the Polymod data on within household contacts to these results to gage how well the studies correspond to each other.

Household demographics probably have changed since the POLYMOD survey was implemented in 2006, and we may see differences reflected in the two data collection methods. (ONS survey report on changing UK demographics: https://www.ons.gov.uk/peoplepopulationandcommunity/populationandmigration/populationestimates/articles/overviewoftheukpopulation/january2021)

Other Editorial Points:

4. Title: Please revise your title according to PLOS Medicine's style. Your title must be nondeclarative and not a question. It should begin with main concept if possible. "Effect of" should be used only if causality can be inferred, i.e., for an RCT. Please place the study design ("A randomized controlled trial," "A retrospective study," "A modelling study," etc.) in the subtitle (ie, after a colon).

**Updated to:** Changes in social contacts as measured by the CoMix contact survey during the COVID-19 pandemic in England between March 2020 and March 2021: a repeated cross-sectional study

5. Data availability statement: PLOS Medicine requires that the de-identified data underlying the specific results in a published article be made available, without restrictions on access, in a public repository or as Supporting Information at the time of article publication, provided it is legal and ethical to do so. Please see the policy at

[http://journals.plos.org/plosmedicine/s/data-availability](http://protect-eu.mimecast.com/s/xDGsCQ7METBj551UYdZSq?domain=journals.plos.org)

and FAQs at

[http://journals.plos.org/plosmedicine/s/data-availability#loc-faqs-for-data-policy](http://protect-eu.mimecast.com/s/XPUPCR1NGcnqAADSLVWl8?domain=journals.plos.org)

Thank you for your willingness to make your data available on the zenodo platform. Please make it clear and provide the link(s) for the repository where the data will be made available. Please include any relevant data identifier, including DOI or accession number.
We will provide this information closer to publication

6. Author list: Please clarify if the individual members of the CMMID COVID-19 working group named in the Acknowledgements are intended to be named as individual authors or not. To qualify for authorship, all contributors must meet at least one of the seven core contributions (conceptualization, methodology, software, validation, formal analysis, investigation, data curation), as well as at least one of the writing contributions (original draft preparation, review and editing). Please see our policy on group authorship here: [https://journals.plos.org/plosmedicine/s/authorship#loc-group-authorship](https://protect-eu.mimecast.com/s/yPdDCVQRMU0oDDnhjx3pM?domain=journals.plos.org)

I will individually add each contributing member if accepted for publication as appropriate.

7. Abstract: Please combine the Methods and Findings sections into one section, “Methods and findings”.

**updated**

8. Abstract: Background: Please provide more context supporting the advance and importance of the study. The final sentence should clearly state the study question.

**updated** to reflect aim of study

“Background

During the COVID-19 pandemic, the UK government imposed public health policies in England to reduce social contacts in hopes of curbing virus transmission. We conducted a repeated cross-sectional study to measure contact patterns weekly in from March 2020 to March 2021 to estimate the impact of these policies in terms of the mean number of contacts, covering three national lockdowns interspersed by periods of lower restrictions.

9. Abstract: Methods and Findings: Please include the study design, population and setting, number of participants, and main outcome measures early on in the description of the Methods.

**Updated**

10. Abstract: Conclusions: Please address the study implications without overreaching what can be concluded from the data; the phrase "In this study, we observed ..." may be useful.

**updated:** In this study, we observed that recorded contacts reduced dramatically compared to pre-pandemic levels (as measured in the POLYMOD study), with changes in reported contacts correlated to government interventions throughout the pandemic.

11. Author Summary: At this stage, we ask that you include a short, non-technical Author Summary of your research to make findings accessible to a wide audience that includes both scientists and non-scientists. The Author Summary should immediately follow the Abstract in your revised manuscript. This text is subject to editorial change and should be distinct from the scientific abstract. Please see our author guidelines for more information: [https://journals.plos.org/plosmedicine/s/revising-your-manuscript#loc-author-summary](https://protect-eu.mimecast.com/s/dhMGCWqVNfzmAALhlpHHB?domain=journals.plos.org)
**Added**

12. Background: Please rename this section as “Introduction” and please expand the Introduction to fully address past research. Please clearly emphasize the novelty, the need for and potential importance of your study. Please conclude the Introduction with a clear description of the study question or hypothesis.

Updated -paragraph2: A key component in the transmission of respiratory viruses is the rate of close social contacts in a population[ref]. Social contact studies have been demonstrated as effective in parameterising mathematical disease models to simulate an outbreak.[[7]](https://paperpile.com/c/1kLfsY/j9lrv) Previous social contact studies have captured contact rates, using a variety of methods including paper diaries, online surveys, and technology capable of recording proximity[[8]](https://paperpile.com/c/1kLfsY/6CmJp). As the Covid-19 pandemic began to spread around the world, several contact studies were conducted to parameterise models during increased risk perception in communities and legal restrictions to social contact[[9]](https://paperpile.com/c/1kLfsY/RxI02). However, no studies, to our knowledge, either during or prior to the Covid-19 pandemic have consistently recorded social contacts in the same population with returning participants over a long period of time, or in combination with participant risk perception and behaviours associated with work, travel, education, and leisure.

paragraph4: While past epidemics have relied on retrospective analyses of the impact of control measures on the course of the epidemic[[11]](https://paperpile.com/c/1kLfsY/4Zh3), the CoMix study has enabled real-time assessment of the impact of control measures on social contacts, one of the main drivers of transmission. The frequency of our surveys have allowed us to capture social contact behaviour under many different social contact policies and during periods of increasing, plateauing, and decreasing covid-19 related hospitalisations. This timely study has enabled rapid feedback for policy interventions to the UK Government, been used as an early indicator of changes or potential changes to the basic reproduction number[[10,12]](https://paperpile.com/c/1kLfsY/uVWXj+HITA), and provides invaluable data to parameterise models and to inform future study of the transmission and control of respiratory diseases.

13. Methods: Please ensure that the study is reported according to the STROBE guideline, or the most appropriate reporting guideline for your study, and include the completed STROBE or other checklist as Supporting Information.

Please add the following statement, or similar, to the Methods: "This study is reported as per the Strengthening the Reporting of Observational Studies in Epidemiology (STROBE) guideline (S1 Checklist)."

The STROBE guideline can be found here: [http://www.equator-network.org/reporting-guidelines/strobe/](http://protect-eu.mimecast.com/s/oq-ICX5WOIBoxxPU2haDi?domain=equator-network.org)

When completing the checklist, please use section and paragraph numbers, rather than page numbers.
**Updated:** All objectives have been met using the STROBE Checklist for cross sectional studies

14. Methods: Did your study have a prospective protocol or analysis plan? Please state this (either way) early in the Methods section. Thank you for including the Protocol as a supporting information file. Please note if this was prospectively developed, and please make sure that the Methods section transparently describes when analyses were planned, and when/why any data-driven changes to analyses took place. Any changes in the analysis-- including those made in response to peer review comments-- should be identified as such in the Methods section of the paper, with rationale.

**Updated:** The original sample size for the UK was 1,500 adults every two weeks, for a total of 16 weeks (8 surveys). With an estimated ~12% dropout rate per wave of interviews, this would have resulted in 500 individuals after the final wave. However, we made the decision to run the survey in two panels running on alternating weeks to make data available on a weekly basis and we topped up the second panel to boost participation. When panels for children’s data were initially recruited, we aimed for approximately 500 participants in the first panel, with panel top ups as needed based on operational analysis needs and recruitment limitations. In August, we recruited two fully new panels that included observations for both adult and children’s data, with an aim for about 3,000 per week with panel top ups. This resulted in continued recruitment of participants for the final panels. The protocol for the study outlined any aims and described multiple analysis techniques to be used over the course of several publications. Some of these aims have already been met, some will be addressed in this study, and some are ongoing. We have added analyses as new study questions and analysis techniques have been identified.

15. Methods: Please specify the nature of informed participant consent. Please clarify if the participants were compensated to participate (this was mentioned in the protocol).
**updated:** Participants agreed to participate in the study under the lawful basis of research in the public interest. All participants demonstrated eligibility by confirming their age, awareness of data privacy protections and the right to decline to participate, and agreement to participate. Participants received compensation for participating for each survey and received a bonus for completing at least 8 waves.

16. Methods: Line 160: Please clarify if these are national hospitalization data for England.

**Updated:** confirmed hospitalisation data is for England only

17. Methods: Please describe how “social grade” was calculated/determined from the survey response.
**Updated:** This is included in the supplementary material - I have updated the text to say “. Participants were assigned social grade based on occupation by the Ipsos-MORI company (see key for social grade categorisation ~~map~~ in the supplementary materials),”

18. Results: Mean contacts: If possible please present the mean daily contacts for study periods in table format, stratified by setting, perceived risk, similar to results presented in Table S3.

Raw results data added as supplementary files.

19. : Line 269 and line 356: We suggest clarifying “during the Christmas period” with the months/dates of the time period instead.
The text in the “study periods section is updated to include the dates, however, we believe that highlighting the social environment of the time period is important, as this was an intervention to mitigate risks associated with this particular holiday, so have decided to continue referring to this as the Christmas period.

20. Discussion: Please present and organize the Discussion as follows: a short, clear summary of the article's findings; what the study adds to existing research and where and why the results may differ from previous research; strengths and limitations of the study; implications and next steps for research, clinical practice, and/or public policy; one-paragraph conclusion. It would be helpful to expand the discussion of the pre-pandemic patterns, to highlight the novelty, advance, and policy implications of the findings presented here and also to provide support for your conclusion that contacts are lower than “normal levels” reported in previous years.

**Updated** (annotated in marked up version of the document)

21. Discussion: Line 389: Please avoid language that implies a causal relationship: “Although easing of restrictions did lead to an increase in contacts, this did not necessarily occur immediately.”

**Updated:** Although we observed an increase in contacts when restrictions were eased, the change did not occur immediately after the policies were implemented.

22. Discussion: Line 421-423: Please clarify and reference the mobility indices mentioned here: “A number of mobility indices such as google and facebook have been made available during the pandemic, which also provide an indication of…” and we suggest capitalizing Google and Facebook.
**updated as suggested**

23. Table 1: It may be helpful, in the Methods or Results, or as supporting information, to have some information on the general characteristics of each lockdown relevant to social contact, in terms of restrictions (for example, what is meant by “relaxed” restrictions, “easing” of restrictions, etc.).

**Updated”** Table added as 1B

24. Table 2: Please provide summary demographic data including part time employment status, perceived risk, income level, and precaution information.
Updated: added data as file to supplementary material

25. Figure 2: It would be helpful to have dates included associated with each lockdown-related study period.

**Updated:** Lockdown period dates are shown in Table 1 - I have added a reference in the text of the results.

26. Table 3: Were any statistical tests done to evaluate changes in relative differences in contacts among age or other groups across study periods?

**Updated**: Added analysis to manuscript and figures to supplementary materials

27. Table S1 and S2: Please clarify if “day of week” is indicated in the table.

**Updated** (does not include day of the week)


Comments from the reviewers:

Reviewer #1: This paper estimates contact patterns over a large period of time during the Covid-19 epidemic in UK. They describe overall changes with respect to pre-pandemic levels and changes between March 2020 and March 2021 due to governmental interventions during the epidemic. They provide a unique dataset on the evolution of contact patterns during the COVID-19 pandemic that can be used to inform future outbreak response and can be applied to transmission of other infectious diseases in similar settings.

Overall, the article is well written, it addresses an important area of research, and contains a lot of work. Considering the need for studies that quantify behavioural changes during a pandemic, this study could have an important contribution to science.

I have some minor points on some part of the Results and the Discussion section.

Pag12, lines 299-306: in its current form the paragraph is confused, and it is not very clear to what they refer by relative differences. Relative with respect to what?

**Updated:** We compare the relative difference of mean contacts using a generalised additive model with Lockdown 1 as the reference period, as this was the beginning of the survey and the period of most stringent lockdown measures. The dates for lockdown periods can be found in Table 1.

Reading the manuscript and with the mounting scientific evidence on the difference in the contribution of indoor/outdoor contacts to SARS-Cov2 transmission, it is quite startling not to see any reference to indoor/outdoor contacts throughout the results and any table reporting contacts by setting even in the supplementary material. If you registered this information, I suggest you discuss it in the paper. Otherwise, I would state this as a quite important limitation of the study and important data to collect in future studies.
**Response:** Although we did include these questions, they were not required. Due to the amount of missing information, we do not include this data in this analysis. Future analyses may explore this further.

Finally, I would discuss better your findings with respect to pre-pandemic observed contact patterns, both in terms of mean number of contacts per person and age specific patterns.

**Updated:** Calculated age specific contact means for the POLYMOD study to provide more perspective on pandemic vs pre-pandemic contacts..


Reviewer #2: This study provides a descriptive analyses showing the mean number of contacts people reported and how these differed during three national lockdowns, periods with more relaxed restrictions, and over the Christmas holiday period.

The study design is well thought through and comprehensive, and the technically appropriate methodology has been clearly and concisely communicated.
The results are fairly presented and accurately interpreted, and the main study limitations have been thoroughly explored in the discussion section.


Reviewer #3: PMEDICINE-D-21-02365R1

Thank you for the opportunity to review the article entitled "CoMix: Changes in social contacts as measured by the 1 contact survey during the COVID-19 pandemic in England between March 2020 and March 2021" (PMEDICINE-D-21-02365R1). This paper provides some extremely useful and relevant information about contact patterns during the past year. This data should be disseminated and the authors have done an excellent and thorough job of explaining the study and presenting the data. However, more context is needed. Right now this reads as a data report, when it is my understanding that the readers of PLOS Medicine will be interested in some context. Perhaps one or two more paragraphs on why this is important and why this data is being reported might help. Some contextual literature on adherence and the importance of social distancing in this pandemic and past pandemics might be relevant (Williams et al., 2020a,b). Also, the main message of the paper, that largely people
are following government guidelines and policy is the main driver of contacts, is somewhat lost in the methodology and data.

I would suggest more of a 'why we care' in the introduction and some emphasis on the main message of the findings in the discussion and abstract prior to publication. That being said, the methodology and results were of an extremely high quality and the CoMix study is excellent. However, readers may not be as knowledgeable about this type of methodology, so clearer contextualization is necessary.

**Updated -** see above in editors comments and paragraphs 2 & 4 in text
