# Supplementary material for: Changes in social contacts in England during the COVID-19 pandemic between March 2020 and March 2021 as measured by the CoMix survey: A repeated cross-sectional study
Source: PLoS Med. 2022 Mar 1;19(3):e1003907. doi: 10.1371/journal.pmed.1003907 (PMC8887739; doi:10.1371/journal.pmed.1003907)
Supplement: Attachment — Submitted filename: PLOS_medicine_response_revision3.docx [file pmed.1003907.s017.docx]

Dear PLOS Medicine Editors and Reviewers,

Thank you for your feedback on the manuscript entitled “CoMix: Changes in social contacts as measured by the contact survey during the COVID-19 pandemic in England between March 2020 and March 2021”. The feedback was valuable, and has improved the quality of the manuscript (see responses below). I have uploaded the figures as .tif files, which pass the check through the PACE tool. Please let me know if these files need further formatting.

Additionally, thank you for acknowledging the contributions of the CMMID working group. However, the work completed by the LSHTM CMMID COVID-19 Working Group has been as a result of regular group discussions, internally shared and discussed analyses, and an internal peer-review process. We are required to submit our work with group authorship in order to reflect our collaborative and iterative processes. The COVID-19 pandemic has prompted unprecedented collaboration within and between university groups, and group authorship on published studies has been approved by several journals, including [PLOS Medicine](https://journals.plos.org/plosmedicine/article?id=10.1371/journal.pmed.1003815).

Examples of CMMID COVID-19 Working Group authorship:
<https://journals.plos.org/plosmedicine/article?id=10.1371/journal.pmed.1003815>

<https://www.thelancet.com/journals/lancet/article/PIIS0140-6736(21)01697-4/fulltext>

<https://www.nature.com/articles/s41591-020-0962-9>

Please consider including the CMMID COVID-19 Working group in the list of authors for this publication. I would be happy to schedule a call to discuss this further or answer any follow-up questions over email.

Best wishes,

Amy

Sincerely,

Amy Gimma

**Responses**

1. Title: We suggest revising slightly: “Changes in social contacts in England during the COVID-19 pandemic between March 2020 and March 2021 as measured by the CoMix contact survey: A repeated cross-sectional study”
**updated**

2. Data availability statement: Thank you for your willingness to share your data. At this time please revise this statement to indicate that all data and code underlying the study is accessible from the three links provided and/or provide all links necessary for interested parties to access the data.
**Updated**

3. Authors: Please include CMMID COVID-19 working group in the author list, and list the individual members of the CMMID COVID-19 working group in the Acknowledgements.

**­Thank you for acknowledging the contributions of the CMMID working group. However, the work completed by the LSHTM CMMID COVID-19 Working Group has been as a result of regular group discussions, internally shared analyses, and an internal peer-review process. We are required to submit our work with group authorship in order to reflect our collaborative and iterative processes. The COVID-19 pandemic has prompted unprecedented collaboration within and between university groups, and group authorship on published studies has been approved by several journals, including** [**PLOS Medicine**](https://journals.plos.org/plosmedicine/article?id=10.1371/journal.pmed.1003815)**.**

**Please consider including the CMMID COVID-19 Working group in the list of authors for this publication. I would be happy to schedule a call to discuss this further or answer any follow-up questions over email.**

**Examples of CMMID COVID-19 Working Group authorship:**
[**https://journals.plos.org/plosmedicine/article?id=10.1371/journal.pmed.1003815**](https://journals.plos.org/plosmedicine/article?id=10.1371/journal.pmed.1003815)

[**https://www.thelancet.com/journals/lancet/article/PIIS0140-6736(21)01697-4/fulltext**](https://www.thelancet.com/journals/lancet/article/PIIS0140-6736(21)01697-4/fulltext)

[**https://www.nature.com/articles/s41591-020-0962-9**](https://www.nature.com/articles/s41591-020-0962-9)

4. Abstract: Line 19: Please consider changing “ lower restrictions” to “fewer restrictions” or similar. **updated**

5. Abstract: Methods and Findings: Please provide more detail on how the survey of the population was implemented (e.g. internet based banner and social media ads, email campaigns).
**updated**

6. Abstract: Line 33-34: We suggest revising to: “Changes in contact patterns were observed over time, and associated with participants’ age, personal risk factors, and perception of risk.”

**updated**

7. Abstract: Line 37-40: Please clarify if this is for adults or children, as contact findings for both age groups were presented above: “This demonstrates a sustained decrease in social contacts compared to a mean of 11.08 (95%CI 10.54 to 11.57) contacts per participant overall as measured by the POLYMOD social contact study in 2005/06.”

**updated**

8. Abstract: Methods and Findings: In the last sentence of the Abstract Methods and Findings section, please describe the main limitation(s) of the study's methodology.
**updated**

9. Abstract: We suggest re-organizing and consolidating the Conclusions section, slightly. We suggest moving the first sentence to the end of the Conclusions, and combining it with the final sentence.
**updated**

10. Abstract: Conclusions: At line 51-53: We suggest revising the sentence to mention the setting: “...the CoMix survey provides a unique repeated cross-sectional data set for the year subsequent to the first lockdown in England…”

**updated**

11. Author summary: We suggest consolidating and reducing the length of each point of the summary. Please consistently use past tense in the section “What did the researchers do and find?”

**updated**

12. Author summary: Why was this study done? Please use “COVID-19” consistently throughout. We suggest revising the first and second points to:
-“Mathematical models can be used to better understand the transmission dynamics of COVID-19, and could be strengthened by empirical evidence of the number of social contacts made under pandemic conditions.”

**Updated**

- “There is a need for real-time social contact data to inform these models, because we predict that behavior will change due to perceived risk and in response to government policies restricting social contact of the course of the panemic, such as during lockdowns.”

**Updated – original reworded**

13. Author summary: What do these findings mean? For the first bullet point, please summarize the implications of the observed decreases in social contact over the first year of the pandemic. We suggest shortening and combining the first three existing bullet points into one point.
**updated**

14. Author summary: We suggest revising the fourth bullet point to “These data may be used by researchers and policymakers to monitor changes…”

**We are aware of ongoing and future plans for both researchers and government officials who are continuing to use this data.**

15. Introduction: Line 123-131: We suggest that this paragraph may be more suited to the Discussion/Conclusions than the Introduction.

**This paragraph was added in the introduction in response to previous reviewers comments. We agree with the previous feedback that this information provides a necessary introduction to the wider CoMix international survey for the reader.**

16. Methods: Ethics statement: Please indicate the nature of informed participant consent. Thank you for indicating that participants agreed to participate, and awareness of right to decline to participate, but please explicitly note participant informed consent to participate, and whether this consent was requested at each survey round.

**Updated – line 149-152**

17. Methods: Please include the completed STROBE checklist as Supporting Information. Please add the following statement, or similar, to the Methods: "This study is reported as per the Strengthening the Reporting of Observational Studies in Epidemiology (STROBE) guideline (S1 Checklist)."

The STROBE guideline can be found here: [http://www.equator-network.org/reporting-guidelines/strobe/](https://protect-eu.mimecast.com/s/U98kCx1g4cxJ1oYsRw2eA?domain=eur03.safelinks.protection.outlook.com)

When completing the checklist, please use section and paragraph numbers, rather than page numbers.

**Uploaded**

18. Methods: Prospective analysis plan: Thank you for your response indicating some changes made to your original study protocol. Please explicitly make note of this in the text, where applicable.

**Updated: Lines 165-185**

19. Results: Line 372-377: If possible it might be helpful to also provide the non-lockdown mean recorded contacts for 0-4 year old children, and also the lockdown mean contacts for 5-17 year old children. This make make the comparisons with the POLYMOD study easier to put into context.
**Updated with reference to the relevant supplementary table - 377**

20. Results: Line 381-413 and 435-453: It may be helpful to illustrate these observations with some quantitative data.

Updated - lines 448-453

21. Discussion: Line 494: Please remove the heading “Summary of Findings”
**Updated**

22. Discussion: Line 504-506: Please revise the wording here to avoid implications of causality (e.g. the use of the word determinant): “The survey results suggest that government action was a major factor in the mean number of social contacts, with contact rates dropping markedly during every lockdown. However, it was not the only determinant.”
**updated – lines 515-521**

23. Discussion: Line 524: Please avoid the term “significantly” here, unless describing a statistical difference.

**updated**

24. Discussion: Line 551: Please consider if “longitudinal” is the best term to describe the survey, or if repeated cross-sectional (or similar) would be accurate.

**Updated - line 580--584**

25. Discussion: Line 554: Please revise to: “ A number of mobility indices such as those implemented by Google and Facebook…”

**Updated – line 552-555**

26. Discussion: Please be sure to organize the Discussion as follows: a short, clear summary of the article's findings (lines 589-626); what the study adds to existing research and where and why the results may differ from previous research (lines 628-681); the strengths and limitations of the study 683-697); the implications and next steps for research, clinical practic9e, and/or public policy (lines 628-681); one-paragraph conclusion. The discussion of the limitations seems as if it should come after the “CoMix in context” section at line 542 (updated). Also, please include a paragraph describing implications for research, practice and policy (this is covered in the conclusion - last sentence) before the Conclusion.

**updated**

27. Figure 6: In the legend, it would be helpful to summarize slightly the key take home point of the contact matrices for each period.

**The summary of the take home point for the matrices is in the text of the document, which is the relative changes in contact patterns by age of both contact and participant throughout the course of the pandemic. Other plots only show changes in contacts based on the participant age, and do not take the age of the contact into account. In this context, there is not a take home point for each individual matrix.**

28. Table 3: Please provide the mean contacts for each age group during each period in a table, in addition to the relative differences (similar to Table S3, but for each of the 9 periods). Also, please note if statistical tests were done to compare differences in contacts during different periods within an age group.

**This data was not calculated for the analysis in this section, as it was provided by week in the section titled “Mean contacts, risk perception, and face coverings.” Statistical uncertainty in the results of the generalised additive model is represented by 95% confidence intervals.**

29. Page 34: Please remove the section “Ethics approval and consent to participate” as this is already included in the Methods section (please be sure all information is complete and accurate). Please remove the sections Consent for Publication, Availability of Data and Materials, Author’s Contributions, Competing Interests, and Funding. Please be sure all information is entered completely in the relevant sections of the manuscript submission system.

**updated**

30. Acknowledgments: Please list the Centre for Mathematical Modelling of Infectious Disease Working Group Authors in the Acknowledgements.

­Thank you for acknowledging the contributions of the CMMID working group. However, the work completed by the LSHTM CMMID COVID-19 Working Group has been as a result of regular group discussions, internally shared analyses, and an internal peer-review process. We are required to submit our work with group authorship in order to reflect our collaborative and iterative processes. The COVID-19 pandemic has prompted unprecedented collaboration within and between university groups, and group authorship on published studies has been approved by several journals, including [PLOS Medicine](https://journals.plos.org/plosmedicine/article?id=10.1371/journal.pmed.1003815).

Please consider including the CMMID COVID-19 Working group in the list of authors for this publication. I would be happy to schedule a call to discuss this further or answer any follow-up questions over email.

Examples of group authorship:
<https://journals.plos.org/plosmedicine/article?id=10.1371/journal.pmed.1003815>

<https://www.thelancet.com/journals/lancet/article/PIIS0140-6736(21)01697-4/fulltext>

<https://www.nature.com/articles/s41591-020-0962-9>

31. References: Please double check formatting of each reference. Please remove the underline for reference 30. Please use the "Vancouver" style for reference formatting, and see our website for other reference guidelines [https://journals.plos.org/plosmedicine/s/submission-guidelines#loc-references](https://protect-eu.mimecast.com/s/eSnZCyXjgiLNr5xhAaeLx?domain=eur03.safelinks.protection.outlook.com)

32. Supporting Information files: Please be sure that each supporting information item has a number and name description. You may use almost any description as the item name of your supporting information as long as it contains an "S" and number. For example, “S1 Appendix” and “S2 Appendix,” “S1 Table” and “S2 Table,” and so forth. Match the names of your supporting information files with the supporting information captions within your manuscript. For example, a PDF file for “S2 Fig.” must be named “S2_fig.pdf”. In the published article, supporting information files are accessed only through a hyperlink attached to the captions. For this reason, you must list captions at the end of your manuscript file. You may include a caption within the supporting information file itself, as long as that caption is also provided in the manuscript file. Do not submit a separate caption file.

33. Supporting Information: Contact Matrices: If possible, please provide brief description of the matrices that facilities understanding by non-specialists who may not be familiar with the interpretation of matrices.

**Updated main text – line 198-104**

34. Table S4: Please reference the POLYMOD study in the legend.

**updated**

35. Figure S1: Please increase the font size used, if possible.

36. Figure S2: Please explain “ABC1” and “C2DE” in the legend.

**updated**

37. Figure S3: Please provide a label for the y axis, if possible.
**updated**

Comments from Reviewers:

Reviewer #3: The authors have addressed the comments comprehensively and I have no further additions/changes.

**Thank you for your comments and for the time invested in reviewing our study.**
